# Supplementary material for: A Degradation Motif in STAU1 Defines a Novel Family of Proteins Involved in Inflammation
Source: Int J Mol Sci. 2022 Sep 30;23(19):11588. doi: 10.3390/ijms231911588 (PMC9569955; doi:10.3390/ijms231911588)
Supplement: Supplementary file 1 [file ijms-23-11588-s001.zip › Supp FIgures IJMS.pdf]

Supplementary figures

Supplementary Figure S1. STAU1^{55} is tagged by K11-ubiquitin chains. HEK293T cells were co-transfected with plasmids coding for STAU1^{55}-HA$_3$ (STAU1) or deletion mutants and for GFP-ubi (+) **(A)** or GFP-ubi-K11R (+) **(B)**. Cells expressing GFP-ubi or GFP-ubi-K11R were further incubated in the presence (+) or absence (-) of MG132 for 8 h. Protein extracts were analyzed by western blotting using anti-HA (to detect STAU1) and anti-GFP (to detect total protein ubiquitination) antibodies. The blots are representative of three independently performed experiments that gave similar results.

Supplementary Figure S2. In silico modelling of the FPL-motif. (A) In silico modelling (I-Tasser software) of the RBD2 domain with the position of amino acids of the FPL-motif. While the predicted alpha helices (α1 and α2) and the beta sheaths (β2 and β3) of RNA-binding domains are conserved, the beta sheath β1 is disordered in the modelling. The FPL-motif is located in the loop between the disordered region and β-sheath 2 (Phe^{39}, Pro^{40}, Pro^{42}, Leu^{45}) and in the beta sheath β2 (Leu^{50}). **(B-M)** Amino acid conservation of the FPL-motif through evolution.
Blue columns: amino acids required for the function of the FPL-motif.
Homo, humans. Macaca, macaques. Rattus, rats. Mus, mice. Bos, cattle. Canis, dog. Xenopus, frogs. Gallus, chicken. Danio, zebrafish.

Supplementary Figure S1

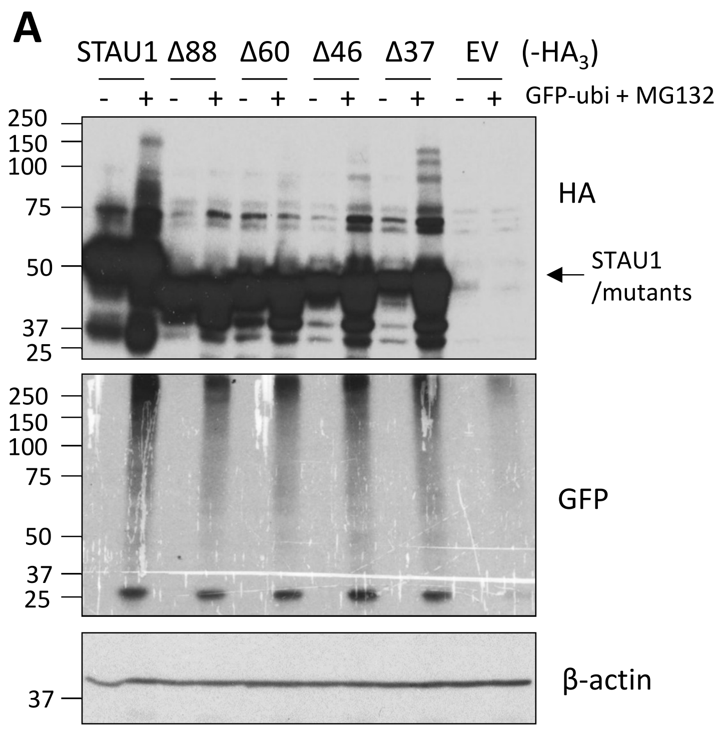

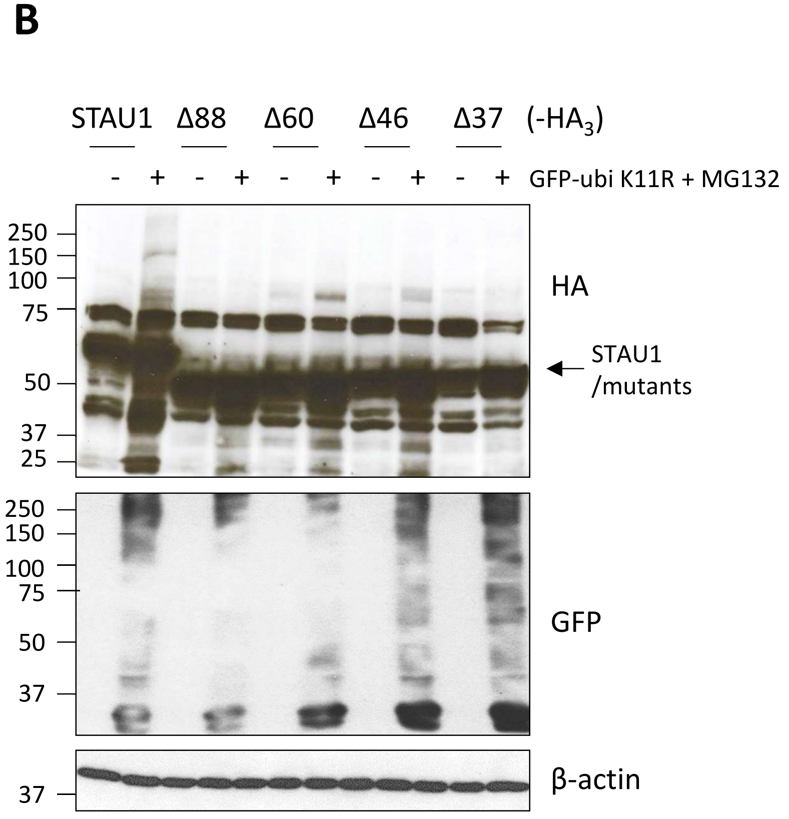

Supplementary Figure S2

A

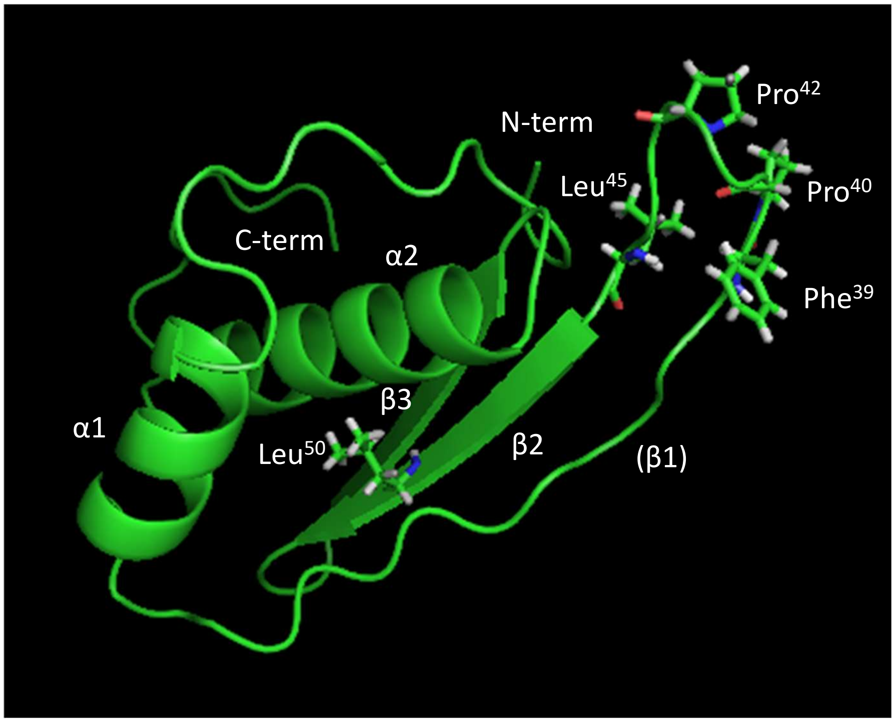

B **STAU1**

Homo	Y	P	F	P	V	P	P	L	L	Y	Q	V	E	L	S
Macaca	Y	P	F	P	V	P	P	L	L	Y	Q	V	E	L	S
Rattus	Y	P	F	P	V	P	P	L	L	Y	Q	V	E	L	S
Mus	Y	P	F	P	V	P	P	L	L	Y	Q	V	E	L	S
Bos	Y	P	F	P	V	p	P	L	L	Y	Q	V	E	L	S
Canis	Y	P	F	P	V	P	P	L	L	Y	Q	V	E	L	S
Xenopus	Y	P	F	P	V	A	P	I	L	Y	Q	V	E	L	S
Gallus	Y	P	F	P	V	G	P	L	L	Y	Q	V	E	L	S
Danio	Y	P	F	PP	V	G	P	V	L	Y	H	M	E	L	S

C **MAP4K1**

Homo	V	L	F	P	L	P	T	P	L	S	V	F	A	L	L
Macaca	V	L	F	P	L	P	T	P	L	S	V	F	A	L	L
Rattus	V	L	F	P	L	P	T	P	L	P	V	F	A	L	L
Mus	V	L	F	P	L	P	T	P	L	P	V	F	T	L	L
Bos	V	L	F	P	L	P	T	P	L	P	V	F	A	L	L
Canis	V	L	F	P	L	P	T	P	L	P	V	F	A	L	L
Xenopus	F	D	F	P	L	P	N	P	L	R	V	F	Q	M	L
Gallus	F	D	F	P	L	P	S	P	L	R	V	F	E	M	L

D **ABCC11**

Homo	P	R	F	P	A	P	Q	P	L	D	N	A	G	L	F
Macaca	P	R	F	P	A	P	Q	P	L	D	D	A	G	L	F
Bos	P	K	F	P	E	P	Q	P	L	D	D	A	G	L	F
Canis	P	K	F	P	A	P	Q	P	M	D	D	A	G	L	F

E **ABCF1**

Homo	F	T	F	P	D	P	P	P	L	S	P	P	V	L	G
Macaca	F	T	F	P	D	P	P	P	L	S	P	P	V	L	G
Rattus	F	T	F	P	D	P	P	P	L	S	P	P	V	L	G
Mus	F	T	F	P	D	P	P	P	L	S	P	P	V	L	G
Bos	F	T	F	P	D	P	P	P	L	S	P	P	V	L	G
Canis	F	T	F	P	N	P	P	P	L	S	P	P	V	L	G
Xenopus	F	T	F	P	N	P	P	P	L	S	P	P	I	L	G
Danio	F	T	F	P	N	P	P	P	L	S	P	P	I	L	G

F **ADGRG1**

Homo	R	S	F	P	D	P	R	G	L	Y	H	F	C	L	Y
Macaca	R	S	F	P	H	P	R	G	L	Y	H	F	C	L	Y
Rattus	Y	F	F	P	E	P	R	G	L	Y	H	F	C	L	Y
Mus	R	F	F	P	E	P	R	G	L	Y	H	F	C	L	Y
Bos	W	P	F	P	L	P	R	G	L	Y	H	F	C	L	Y
Canis	Q	L	F	P	E	P	R	G	L	Y	H	F	C	L	Y
Xenopus	F	T	L	Q	S	L	P	G	P	Y	I	F	C	V	H
Gallus	Y	S	L	P	T	T	L	G	R	Y	R	F	C	I	Y
Danio	L	D	S	N	P	H	Q	N	Q	S	H	F	C	V	F

G **GPR83**

Homo	P	D	F	P	E	P	A	D	L	F	W	K	Y	L	D
Macaca	P	D	F	P	E	P	A	D	L	F	W	K	Y	L	D
Rattus	P	D	F	P	E	P	A	D	L	F	W	K	Y	L	D
Mus	P	D	F	P	E	P	A	D	L	F	W	K	Y	L	D
Bos	P	D	F	P	E	P	A	D	L	F	W	K	Y	L	D
Canis	P	D	F	P	E	P	A	D	L	F	W	K	Y	L	D
Xenopus	P	D	F	P	E	P	S	D	L	F	W	K	Y	L	D
Gallus	P	D	F	P	E	P	A	D	L	F	W	K	Y	L	D
Danio	P	S	F	P	H	P	S	D	L	F	W	K	Y	L	D

H **KBTBD13**

Homo	A	G	F	P	R	P	G	S	L	Q	T	F	L	L	R
Macaca	A	G	F	P	R	P	G	S	L	Q	T	F	L	L	R
Rattus	A	G	F	P	R	P	G	S	L	Q	T	F	L	L	R
Mus	A	G	F	P	R	P	G	S	L	Q	T	F	L	L	R
Bos	A	G	F	P	R	P	G	S	L	Q	T	F	L	L	R
Canis	A	G	F	P	R	P	G	S	L	Q	T	C	L	L	R
Xenopus	A	G	F	N	R	G	G	S	L	H	T	F	F	L	R
Gallus	S	E	F	P	S	P	H	Q	L	R	Y	D	V	R	L
Danio	T	G	F	P	R	I	G	S	K	W	T	F	L	L	R

I **LINGO4**

Homo	T	A	F	P	S	P	D	K	L	V	T	L	R	L	S
Macaca	T	A	F	P	S	P	D	K	L	V	T	L	R	L	S
Rattus	T	A	F	P	S	P	D	K	L	V	T	L	R	L	S
Mus	T	A	F	P	S	P	D	K	L	V	T	L	R	L	S
Bos	T	A	F	P	S	P	D	K	L	V	T	L	R	L	S
Canis	T	A	F	P	S	P	D	K	L	V	T	L	R	L	S
Xenopus	D	A	L	P	S	P	L	G	L	E	T	L	L	L	S

J **MCHR2**

Homo	F	F	F	P	L	P	L	I	L	V	C	Y	I	L	I
Macaca	F	F	F	P	L	P	L	I	L	V	C	Y	I	L	I
Bos	S	F	F	P	L	P	L	I	L	M	C	Y	I	L	I
Canis	F	F	F	P	L	P	L	I	L	V	C	Y	I	L	I
Xenopus	F	F	F	P	L	P	L	I	L	A	C	Y	I	L	I
Gallus	F	V	I	P	V	L	V	I	S	L	S	Y	T	R	T

K **OMD**

Homo	F	P	F	P	L	P	K	S	L	E	R	L	L	L	G
Macaca	F	P	F	P	L	P	K	S	L	E	R	L	L	L	G
Rattus	F	P	F	P	L	P	K	S	L	E	R	L	L	L	G
Mus	F	P	F	P	L	P	K	S	L	E	R	L	L	L	G
Bos	F	P	F	P	L	P	K	S	L	E	R	I	F	L	G
Canis	F	P	F	P	L	P	K	S	L	E	R	L	L	L	G
Xenopus	I	P	P	D	L	P	S	S	V	E	R	L	N	F	A
Gallus	F	P	F	P	L	P	S	S	L	E	R	L	L	L	G
Danio	I	P	S	P	L	P	K	T	L	K	R	L	H	L	G

Supplementary Figure S2

L **UBE2F**

Homo	V	H	F	P	D	P	N	K	L	H	C	F	Q	L	T
Macaca	V	H	F	P	D	P	N	K	L	H	C	F	Q	L	T
Rattus	V	H	F	P	D	P	N	K	L	H	C	F	Q	L	T
Mus	V	H	F	P	D	P	N	K	L	H	C	F	Q	L	T
Bos	V	H	F	P	D	P	N	K	L	H	C	F	Q	L	T
Canis	V	H	F	P	D	P	N	K	L	H	C	F	Q	L	T
Xenopus	V	N	F	P	D	P	N	K	L	H	Y	F	H	L	T
Gallus	V	N	F	P	D	P	N	K	L	H	Y	F	Q	L	T
Danio	V	T	F	P	D	E	N	K	L	C	H	F	Q	L	A

M **VCAM1**

Homo	Q	G	F	P	A	P	K	I	L	W	S	R	Q	L	P
Macaca	H	G	L	P	A	P	K	I	L	W	S	R	Q	L	P
Rattus	D	G	F	P	T	P	K	I	L	W	S	R	Q	L	K
Mus	D	G	I	P	A	P	K	I	L	W	S	R	Q	L	N
Bos	N	G	L	P	A	P	K	I	L	W	S	R	K	L	S
Canis	D	G	L	P	A	P	K	I	L	W	S	R	R	L	S
Xenopus	E	A	F	P	T	P	T	L	I	L	K	E	K	T	E
Gallus	D	S	N	P	P	A	Q	V	F	W	R	K	H	L	A
Danio	D	S	F	P	A	G	R	M	V	L	S	R	V	V	D
