# Supplementary material for: A Degradation Motif in STAU1 Defines a Novel Family of Proteins Involved in Inflammation
Source: Int J Mol Sci. 2022 Sep 30;23(19):11588. doi: 10.3390/ijms231911588 (PMC9569955; doi:10.3390/ijms231911588)
Supplement: Supplementary file 1 [file ijms-23-11588-s001.zip › Supp Table S7 IJMS.pdf]

Supplementary Table S7

List of oligonucleotides used in this study

**Plasmids**	**Oligonucleotide primers**	
	Sense	Antisense
Y37A	5’-GGTACTTT**GC**CCCATTTCC-3’	5’-GGAAATGGG**GC**AAAGTACC-3’
P38A	5’-GTACTTTTAC**G**CATTTCCAGTTCC-3’	5’-GGAACTGGAAATG**C**GTAAAAGTAC-3’
F39A	5’-CTTTTACCCA**GC**TCCAGTTCCACC-3’	5’-GGTGGAACTGGA**GC**TGGGTAAAAG-3’
P40A	5’-CTTTTACCCATTT**G**CAGTTCCACC-3’	5’-GGTGGAACTG**C**AAATGGGTAAAAG-3’
V41A	5’-CCCATTTCCAG**C**TCCACCTTTAC-3’	5’-GTAAAGGTGGAG**C**TGGAAATGGG-3’
P42A	5’-CCCATTTCCAGTT**G**CACCTTTAC-3’	5’-GTAAAGGTG**C**AACTGGAAATGGG-3’
P43A	5’-CATTTCCAGTTCCA**GC**TTTACTTTATC-3’	5’-GATAAAGTAAAG**C**TGGAACTGGAAATG-3’
L44A	5’-CAGTTCCACCT**GC**CACTTTATCAAG-3’	5’-CTTGATAAAGT**GC**AGGTGGAACTG-3’
L45A	5’-CAGTTCCACCTTTA**GC**TTATCAAGTG-3’	5’-CACTTGATAA**GC**TAAAGGTGGAACTG-3’
Y46A	5’-CCACCTTTACTT**GC**TCAAGTGGAAC-3’	5’-GTTCCACTTGA**GC**AAGTAAAGGTGG-3’
N47A	5’-CTTTACTTTAT**GC**AGTGGAAC-3’	5’-GTTCCACT**GC**ATAAAGTAAAG-3’
V48A	5’-CTTTATCAAG**C**GGAACTTTCTG-3’	5’-CAGAAAGTTCC**G**CTTGATAAAG-3’
E49A	5’-TATCAAGTGG**C**ACTTTCTGTGG-3’	5’-CCACAGAAAGT**G**CCACTTGATA-3’
L50A	5’-CAAGTGGAA**GC**TTCTGTGGG-3’	5’-CCCACAGAA**GC**TTCCACTTG-3’
S51A	5’-GTGGAACTT**G**CTGTGGGAGG-3’	5’-CCTCCCACAG**C**AAGTTCCAC-3’
RBD2	5'-TACCGAATTCATGTATAAGCCTGTTGACCCTTAC-3'	5'-TACCACCGGTGATTCTCTTCCATTCACCTCCAG-3'
RBD2 $^{\Delta51}$	5’-TACCGAATTCATGGTGGGAGGACAGCAATTTAATG-3’	5'-TACCACCGGTGATTCTCTTCCATTCACCTCCAG-3'
RBD2 $^{\Delta46}$	5’-TACCGAATTCATGCAAGTGGAACTTTCTGTGGG-3’	5'-TACCACCGGTGATTCTCTTCCATTCACCTCCAG-3'
RBD2 $^{\Delta37}$	5’-TACCGAATTCATGCCATTTCCAGTTCCACCTTTAC-3’	5'-TACCACCGGTGATTCTCTTCCATTCACCTCCAG-3'
MAP4K1	5’-CTCGAGATGGACGTCGTGGACCCTGAC-3’	5’-<br>ATTCATAAGCGGCCGCTCCTGGATGTAGAGGTTG-3’
MAP4K1^{F651A}	5’-GCAGGTGCTGGCCCCACTGCCG-3’	5’-CGGCAGTGGGGCCAGCACCTGC-3’
TRIM25	5’-GGAGGGAGACTTTCAGTCCTTGAC-3’	5’-ATAGGTCAGGCTGACTCAAAGCC-3’
TRIP12	5’-GTTTCATCTGTACTTGACATTTTATCC-3’	5’-GTCCCGTGTATTGAAGGAGG-3’
ZNF598	5’-TGCTGGGACCTGTTGTTGC-3’	5’-CACAACACATCTGTGACATCTGACT-3’
STAU1^{55}-HA$_3$-BioID2	5-ATATATGAGCTCATGAAACTTGGAAAAAAACCAATG-3	5’-ATATATAGCGCTGGCACCTCCCACACACAGAC-3
STAU1^{55}-HA$_3$-TurboID	5’-TAAGCAGCGGGCCGCGCCACCATGAAACTTGGAAAAA AACCA-3’	5’-TGTACAGCTAGCGAATTCGAATCCGGAGACGTA CGATGCGTAATCCGGTACATC-3’
HPRT	5’-GCTTTCCTTGGTCAGGCAGAT-3’	5’-CTTCGTGGG GTCCTTTTCACC-3’
RPL22	5’-TTGCTGTTAGCAACTACGCGCAAC-3’	5’-TGGTGACCATCGAAAGGAGCAAGA-3’
